# Decrease in Heparan Sulphate Binding in Tropism-Retargeted Oncolytic Herpes Simplex Virus (ReHV) Delays Blood Clearance and Improves Systemic Anticancer Efficacy

**DOI:** 10.3390/cancers16061143

**Published:** 2024-03-13

**Authors:** Andrea Vannini, Federico Parenti, Cristina Forghieri, Gaia Vannini, Catia Barboni, Anna Zaghini, Tatiana Gianni, Gabriella Campadelli-Fiume

**Affiliations:** 1 Department of Medical and Surgical Sciences, University of Bologna, 40126 Bologna, Italy; andrea.vannini5@unibo.it (A.V.); federico.parenti5@unibo.it (F.P.); cristina.forghieri@unibo.it (C.F.); gaia.vannini@studio.unibo.it (G.V.); 2 Department of Pharmacy and Biotechnology, University of Bologna, 40126 Bologna, Italy; 3 Department of Veterinary Medical Sciences, University of Bologna, 40126 Bologna, Italy; catia.barboni@unibo.it (C.B.); anna.zaghini@unibo.it (A.Z.)

**Keywords:** oncolytic virus, oncolytic herpes simplex virus, retargeting, HER2, heparan sulphate, systemic oncolytic therapy, oncolytic virus biodistribution, gC, glycosaminoglycans, chondroitin sulphate

## Abstract

**Simple Summary:**

Oncolytic herpes simplex viruses (oHSVs) employ as natural entry receptors nectin1 or HVEM. In contrast, retargeted oHSVs (ReHVs) employ as an entry receptor a tumor-associated antigen that no longer interacts with nectin1/HVEM and, thus, spares normal cells. Prior to entry, both oHSVs and ReHVs attach to cells by interaction of gC and gB glycoproteins with heparan sulphates and chondroitin sulphates (glycosaminoglycans–GAGs). The systemic delivery of oncolytic viruses would be the ideal route for hard-to-reach or metastatic cancers that constitute unmet clinical needs. In an accompanying paper, we reported that the blood complement and the antiviral neutralizing antibodies represent the major blood factors that inactivate systemically administered ReHVs. Here, we asked whether ReHV adsorption to GAGs acts as a sink and subtracts the virus from circulation. We report that a genetic modification in gC, which reduced its interaction with GAGs, resulted in a longer half-life of circulating ReHV and a higher anticancer efficacy of systemically (but not intratumorally) administered ReHV.

**Abstract:**

The role of the interaction with cell-surface glycosaminoglycans (GAGs) during in vivo HSV infection is currently unknown. The rationale of the current investigation was to improve the anticancer efficacy of systemically administered retargeted oHSVs (ReHVs) by decreasing their binding to GAGs, including those of endothelial cells, blood cells, and off-tumor tissues. As a proof-of-principle approach, we deleted seven amino acids critical for interacting with GAGs from the glycoprotein C (gC) of R-337 ReHV. The modification in the resulting R-399 recombinant prolonged the half-life in the blood of systemically administered R-399 and enhanced its biodistribution to tumor-positive lungs and to the tumor-negative liver. Ultimately, it greatly increased the R-399 efficacy against metastatic-like lung tumors upon IV administration but not against subcutaneous tumors upon IT administration. These results provide evidence that the increased efficacy seen upon R-399 systemic administration correlated with the slower clearance from the circulation. To our knowledge, this is the first in vivo evidence that the partial impairment of the gC interaction with GAGs resulted in a prolonged half-life of circulating ReHV, an increase in the amount of ReHV taken up by tissues and tumors, and, ultimately, an enhanced anticancer efficacy of systemically administered ReHV.

## 1. Introduction

Oncolytic viruses (OVs) are being developed as therapeutic agents to combat cancers. They are in the preclinical and clinical stages of development [1,2,3,4,5,6,7]; their anticancer efficacy can be augmented when combined with checkpoint inhibitors [8,9,10,11]. Two of the four OVs that received clinical approval are derivatives of the herpes simplex virus (HSV); they are named OncoVEX^GM-CSF^, or T-VEC (commercial name talimogene laherparepvec, or Imlygic), and G47Δ (commercial name Delytact) and are employed against cutaneous melanoma and glioblastoma multiforme, respectively [12,13,14,15,16]. Our laboratory has generated OVs with a specific tropism for cancer cells. They are detargeted from the natural tropism by the deletion of glycoprotein D (gD) residues critical for the interaction with the natural entry receptors nectin1 and Herpesvirus Entry Mediator (HVEM), and retargeted to selected cancer cells by insertion in gD of a single-chain antibody to the cancer-specific antigen of choice, which becomes the new virus receptor [17,18]. In preclinical models, the resulting retargeted herpesviruses (ReHVs) showed high specificity and a high safety profile, as well as high anticancer efficacy [19,20,21,22].

The approved OVs and the majority of those in clinical trials are administered intratumorally. Nonetheless, systemic administration would be desirable for the hard-to-reach and the metastatic cancers that constitute unmet clinical needs and the leading cause of cancer-related death. Even the OVs that, in the clinical setting, emerged as the best performers for systemic administration have led to substantially debated or unsatisfactory results [23,24,25,26]. Several factors mar the systemic administration of OVs, including antiviral factors in the blood (the complement) that inactivate the circulating virus, the nonspecific adsorption to off-target tissues, the risk of off-tumor and off-target infections due to the widespread distribution of OV receptors, and specific immunity (e.g., neutralizing antibodies) to OV [27,28,29,30,31,32]. The latter applies to OVs derived from seroprevalent human viruses and OVs derived from animal viruses when administered repeatedly [30,33,34]. In a paper published in the current special issue, our group reported on the systemic delivery of the HER2-retargeted ReHV named R-337, its blood clearance and biodistribution, and its anticancer efficacy against lung cancer, a model of metastatic disease [22].

A feature that has received little if any attention is the contribution to the clearance of systemically administered OVs by virus adsorption to off-tumor and off-target tissues. A large number of viruses attach to and enter cells by a stepwise process. The initial attachment often occurs to broadly expressed unspecific molecules exemplified by glycosaminoglycans (GAGs), in particular, heparan sulphates (HSs) and chondroitin sulphates (CSs) [35,36,37,38,39,40]. This step is followed by an interaction with more specific receptors that determine the tropism of a particular virus and mediate its entry into the cell. In the case of HSV, the attachment receptors are GAGs, specifically HSs and CSs, that interact with the virion glycoproteins C (gC), and, to a minor extent, gB [41,42,43,44,45]. Attachment is followed by the interaction of gD with the entry receptors nectin1 or HVEM (herpesvirus entry mediator) and the subsequent engagement of further receptors like integrins and gB receptors [46,47]. The site in gC that interacts with GAGs has been finely mapped [48,49].

Despite numerous studies that dealt with the contribution of HSV gC to the interaction with cell surface HSs and CSs within in vitro cultured cells, the role of the HSV interaction with GAG molecules during in vivo infection is currently unknown. We hypothesized that the virion–GAG interaction might act as a sink for systemically administered oHSVs, including ReHVs, and subtract them from circulation. Subtraction would occur regardless of whether the tissues to which the virus attaches promote virus entry and productive infection, or whether they capture the virus and target it for degradation. Cell candidates for such subtractions include endothelial cells lining blood vessels whose luminal faces are highly enriched in HSs, cells that form the glycocalyx [50], and some of the circulating blood cells, as well as other tissues.

The aim of the current study was to investigate whether the anticancer efficacy of systemically delivered ReHVs can be improved by genetic modifications—in particular, by mutations in gC that decrease ReHV binding to GAGs. Such modifications would add to the lack of binding to nectin1, an intercellular molecule highly represented in blood vessels [51,52]; the latter feature is intrinsic to ReHVs and likely further reduces the adhesion to endothelial cells and blood cells. We expected that the combination of decreased binding to GAGs and the lack of binding to the natural HSV entry receptors would increase the half-life of circulating ReHV, and that the longer persistence in blood would also increase the amount of virus that ultimately reaches tissues and specific cancer targets. To provide proof-of-principle evidence, we deleted from R-337 gC a sequence that contains seven amino acid residues (Arg143, Arg145-Thr150) critical for interaction with HSs and CSs [48,49]. We left gB unmodified since this glycoprotein is not the major HS-binding moiety in HSV [44] and its genetic modifications often lead to defects in its fusogenic activity, which is an essential step in HSV entry. The resulting recombinant, named R-399, was characterized in vitro and in vivo upon systemic and intratumoral delivery. We report that R-399 indeed exhibited a longer half-life in blood and higher systemic anticancer efficacy than the parental R-337.

## 2. Materials and Methods

### 2.1. Cells and Viruses

SK-OV-3 human ovarian cancer cells (Roswell Park Memorial Institute), CT26 murine colon carcinoma cells (both cells from American Type Culture Collection), and their derivatives were cultured in RPMI-Glutamax (Thermo Fisher Scientific, Waltham, MA, USA) supplemented with 10% fetal bovine serum (FBS) (Thermo Fisher Scientific). HEp-2 human hepatocellular carcinoma cells (obtained from Dr. B. Roizman, University of Chicago) were cultured in Dulbecco’s Modified Eagle’s Medium (DMEM, Thermo Fisher Scientific) supplemented with 5% FBS. R-337 was described [53], and R-399, which harbors Arg143 and Arg145-Thr150 deletions in gC, was obtained from BAC-337 by galK recombineering [54,55]. For the first recombination, the galK cassette, with homology arms to gC, was amplified with oligonucleotides gC_HSBD_galKF CTAAACCCCCCGGGCCCGTGTGGTGCGACCGCCGCGATTTATTGGCCCGGCCTGTTGACAATTAATCATCGGCA and gC_HSBD_galKR ATTGGGGGGGACGGACCCATGGAGTAACGCCATATCTGGAGGCGGAACTCTCAGCACTGTCCTGCTCCTT, using pGalK as the template. The DNA fragment for the second recombination was generated through the annealing and extension of oligonucleotides gC_del_con_Cfor GGTGCGACCGCCGCGATTTATTGGCCCGGTACGGCTCGCGGGTGCAGATCTGCCGCATGGAGTTC, and gC_del_con_Crev GGGGACGGACCCATGGAGTAACGCCATATCTGGAGGCGGAACTCCATGCGGCAGATCTGCAC. R-399 was reconstituted in SK-OV-3 cells, single plaque-purified, and checked by sequencing. Both R-337 and R-399 viruses were cultured in SK-OV-3 cells.

### 2.2. Virus Adsorption Assay

To assess virus adsorption to cells in the presence of a competitor for virus-GAG binding, 150 PFU of R-337 or R-399 were incubated, in triplicate, with increasing amounts of low molecular weight (LMW) heparin (0.6 μg/mL; 1.8 μg/mL, 5.5 μg/mL, 16.6 μg/mL, 50 μg/mL; Merck KGaA, Darmstadt, Germany; catalog number: 1162487) for 1 h at 4 °C or left untreated, according to [56]. The virion/heparin mixtures were then allowed to adsorb to SK-OV-3 cells for 2 h at 4 °C. After removal of the inoculum, the cells were rinsed three times with cold PBS, shifted to 37 °C for 1 h to allow for penetration of the adsorbed virus, and subsequently overlaid with medium containing 1% of agar. Plaques were scored 3 days later. Results are reported as the percentage of plaques formed relative to those formed in the heparin-untreated control sample [56]. The experiment was repeated three times. To determine the genome copies (GC) to PFU ratio in the purified virion stocks, GC was quantified by qPCR of the viral DNA polymerase gene, herein HSV DNApol, as detailed in [21]. A typical qPCR reaction contained a 1:1000 dilution of purified DNA, 5 μL of TaqMan Fast Advanced Master Mix (Thermo Fisher Scientific; Catalog number: 4444557) and 0.5 μL of the HSV DNApol oligonucleotides, in a final volume of 10 μL. The qPCR reactions were carried out in triplicates in a StepOnePlus system (Applied Biosystems, Waltham, MA, USA) following the protocol of the Master Mix. The mean Ct values were interpolated on a standard curve, which were prepared using seven serial 1:10 dilutions of purified genomic HSV DNA (20–20,000,000 genome copies per reaction) [21]. Standard curve equation was Log (HSV genome copies) = −0.2931 × Ct + 12.16; R² = 0.997. Viral genome copies were expressed as GC/mL. The GC/PFU ration was then calculated.

To assess the virus adsorption to cells displaying decreasing amounts of GAGs, triplicate monolayers of HEp-2 cells were pre-treated with serial dilutions of heparinase I (Merck KGaA; Catalog number: H2519), ranging from 2 to 1000 mU/mL for 1 h at 37 °C or untreated. The cells were then cooled for 30 min at 4 °C, washed with cold PBS and overlaid for 1 h at 4 °C with DMEM containing 5% FBS. R-337 or R-399 viruses (10 PFU/cell) were added to medium and incubated for 1 h at 4 °C with continuous shaking to allow for adsorption. Cells were then washed to remove unadsorbed virions; total DNA was purified by means of Wizard SV Genomic DNA Purification System (Promega Corporation, Madison, WI, USA; Catalog number: A2361). HSV genome copies were quantified by qPCR HSV DNApol. The qPCR reaction was as described in the preceding paragraph, except that the templated DNA was 50 ng of the DNA preparations and the human SOCS1 probe (Thermo Fisher Scientific; Hs00705164_s1) was included. The SOCS1 probe targeted a sequence within the cellular genomic DNA (amplified sequence aligned to a single exon). The ΔCt HSV DNApol-SOCS1 values were calculated to compensate for small differences in the theoretical 50 ng of input DNA. For each virus, ΔΔCt values were calculated with respect to the corresponding control sample consisting of R-337 or R-399 virus adsorbed to cells untreated with heparinase; fold change values were calculated and expressed as percentage values relative to the control samples, made as 100%.

### 2.3. Efficiency of Infection, Virus Growth, Plaque Formation, and Cytotoxicity Assay

To determine the efficiency of R-399 or R-337 infection of SK-OV-3 and CT26-HER2 cells (plating efficiency), 5 × 10^8^ PFU were 1:10 serially diluted and plated in triplicate on the indicated cell lines. After 90 min, the virus inoculum was removed, the infected cultures were overlaid with medium containing 1% agar (SeaPlaque Agarose, Lonza, Rockland, ME, USA), and the number of plaques was scored 5 days later. The experiment was repeated twice. For plaque-size determination, the area of 20 plaques from the plating efficiency experiment was determined for each virus by means of Nis Elements–AR-Imaging Software (Version 2.30, Nikon–Nikon Europe BV–Italy Branch, Florence, Italy). To quantify the extent of virus replication, triplicate monolayers of SK-OV-3 or CT26-HER2 cells were infected at an input multiplicity of infection of 0.1 PFU/cell; unadsorbed virus was removed after 90 min. The non-penetrated virus was inactivated by means of acidic wash (40 mM citric acid, 10 mM KCl, 135 mM NaCl, pH 3.0) (Merck KGaA). Replicate cultures were frozen at the indicated times after infection. The progeny virus was titrated in triplicate in SK-OV-3 cells, and plaques were scored 5 days later. The experiment was repeated twice. For the cytotoxicity assay, SK-OV-3 and CT26-HER2 cells were seeded in 96 well plates (8 × 10^3^ cells/well) in quadruplicate and infected at an input multiplicity of infection of 0.1 PFU/cell. AlamarBlue Cell Viability Reagent (Life Technologies-Thermo Fisher Scientific, Waltham, MA, USA; Catalog number: DAL1025) was added to the culture media (10 μL/well) at the indicated times after infection and incubated for further 4 h at 37 °C. Plates were read at 560 nm and 600 nm with GloMax Discover System (Promega Corporation). Cytotoxicity was expressed as the percentage of non-viable cells relative to uninfected viable cells (alamar blue reduction). The background value (medium alone) was subtracted from each sample. The experiment was repeated twice. To quantify murine IL-12 secretion from infected cells, 6 × 10^4^ SK-OV-3 or 4 × 10^5^ CT26-HER2 cells were infected in triplicate at an input multiplicity of infection of 1 PFU/cell; mIL-12 (p70) concentration was quantified at the indicated time points by means of the Mouse IL-12 ELISA kit (R&D Systems, Minneapolis, MN, USA. Catalog number: M1270).

### 2.4. R-337 and R-399 Biodistribution and Replication in Mouse Tissues

To determine the biodistribution to selected tissues of IV-administered R-337 or R-399, total DNA was purified from a few mg of tissue homogenates by means of Wizard SV Genomic DNA Purification System (Promega Corporation; Catalog number: A2361); the number of viral genome copies in the DNA samples was quantified by qPCR HSV DNApol, as detailed in the Virus Adsorption assay. The mouse Rpl13a (Thermo Fisher Scientific; Mm01612987_g1) was substituted for the human SOCS1 probe. The Rpl13a probe targeted a sequence within murine genomic DNA (the amplified sequence aligned to a single exon) and its Ct values were used to compensate for minimal differences in the input DNA. Specifically, a mean Ct value (hereafter, the reference Ct) of Rpl13a was calculated considering all samples. The ∆Ct was calculated as the difference between the Ct of the sample versus the reference Ct. These ∆Ct values were subtracted from the Ct values of HSV DNApol, obtaining the corrected Ct values that were interpolated on a standard curve, prepared using seven serial 1:10 dilutions of purified genomic HSV DNA (20–20,000,000 genome copies per reaction) [21]. Standard curve equation was Log (HSV genome copies) = −0.2931 × corrected Ct + 12.16; R² = 0.997. Viral genome copies were expressed as GC/100 ng of DNA.

To determine the kinetics of R-337 and R-399 clearance from blood, blood samples were obtained from mice and immediately mixed with 2.5 mM EDTA to prevent clotting. Samples were serially diluted and plated on SK-OV-3 cells. After 90 min, the infected cultures were overlaid with 1% agar-containing medium; the number of plaques was scored 5 days later.

### 2.5. In Vivo Experiments

BALB/c mice transgenic for, and tolerant to, human HER2 (BALB/c-TG) were previously described [57] and bred in the facility of the Department of Veterinary Medical Sciences, University of Bologna. The animals were housed in individually ventilated cages measuring 500 cm² each (NexGen™ Mouse 500, Allentown Inc., Allentown, NJ, USA), with 50 air changes per hour via HEPA-filtered air and had access to feed and water ad libitum. Four-to-six conspecifics were housed per cage to promote species-specific behavior. Enrichment materials such as cardboard tunnels and sizzle nests were provided to encourage natural behaviors. The housing facility is equipped with temperature (20–24 °C) and relative humidity (45–65%) control and features air exchange (15–20 changes per hour) and a 12 h day–night cycle.

Both male and female mice, 10–12-weeks old and weighing 23–25 g, were employed for the experiments in accordance with the ethics committee’s request. The animals were observed daily by monitoring their general condition and the growth of induced SC tumors, as well as carefully assessing any changes in respiration attributable to the metastatic lung masses. This monitoring included meticulous observation of posture, locomotion, size, and condition of the SC tumor masses. At the end of the experiments, the animals were sacrificed by gaseous overdose of isoflurane (Iso–Vet, Piramal Critical Care, Voorschoten, The Netherlands) followed by cervical dislocation, after which blood and tissue were collected for subsequent analysis.

For intravenous (IV) therapy of metastatic tumors, 3 × 10^5^ CT26-HER2 cells were administered via an IV injection into the tail vein of 10–12-week-old BALB/c-TG mice in 100 μL of serum-free medium. On Days 11 and 15, R-337 and R-399 (4 × 10^7^ PFU/injection) were administered IV in 100 μL of Phosphate Buffer Saline (PBS) or vehicle (PBS alone). At sacrifice (Day 25), lungs were harvested, perfused with the staining solution (PBS with 15% India ink; Pebeo, Gémenos-Cedex, Gémenos, France; Catalog number: 13420) and fixed in Fekete’s solution (61% ethanol, 32% water, 4% acetic acid and 3% formaldehyde). Superficial lung nodules were counted under the microscope as white spots. For intratumor (IT) therapy of subcutaneous tumors, 1 × 10^6^ CT26-HER2 cells were implanted subcutaneously (SC) in the flank of BALB/c-TG mice in 100 μL of serum-free medium. Tumor volumes were measured two times a week by measuring the major and minor diameters and calculating the volumes by the formula: major diameter × (minor diameter)^2^ × 0.5. Twenty days after tumor grafting, when tumor volume averaged 70-100 mm^3^, mice received 3 IT injections of R-337 or R-399 (3 × 10^7^ PFU for each injection in 100 µL of PBS) or vehicle (PBS alone), at 3–4-day intervals. Mice were sacrificed when their tumors reached a volume of about 1500 mm^3^, ulceration occurred, or the animals showed distress or pain. For biodistribution experiments, 1 × 10^6^ CT26-HER2 cells were administered via an IV injection into the tail vein of BALB/c-TG mice. After 14 days, mice received an IV injection of R-337 or R-399 (1 × 10^7^ PFU in 100 µL of PBS). Mice were sacrificed at the indicated times and blood and tissue were collected. Determination of splenocyte reactivity and serum antibodies to cancer cells were performed as described [22].

### 2.6. Statistical Analysis

All statistical analyses were performed with GraphPad Prism Version 8.4.3. For each comparison, the Shapiro–Wilk test was used to check the normal distribution of the results. Where normal distribution was confirmed, F-test or Bartlett’s test were used to check the equality of variances between the populations. If the variances were equal, the data were compared with either the unpaired parametric *t*-test (two-tailed) or the ordinary one-way ANOVA with Tukey’s correction (all possible comparisons between the study groups were considered); if the variances were different, the unpaired parametric *t*-test (two-tailed) with Welch’s correction was employed. If the results did not follow a normal distribution, we employed either the unpaired nonparametric Mann–Whitney test (two-tailed) or the nonparametric Kruskal–Wallis test with Dunn’s correction (all possible comparisons between the study groups were considered).

## 3. Results

### 3.1. Engineering of a HER2-Retargted Oncolytic HSV Partially Deleted in Heparan Sulphate Binding Activity (R-399): In Vitro Properties

**Figure 1:**
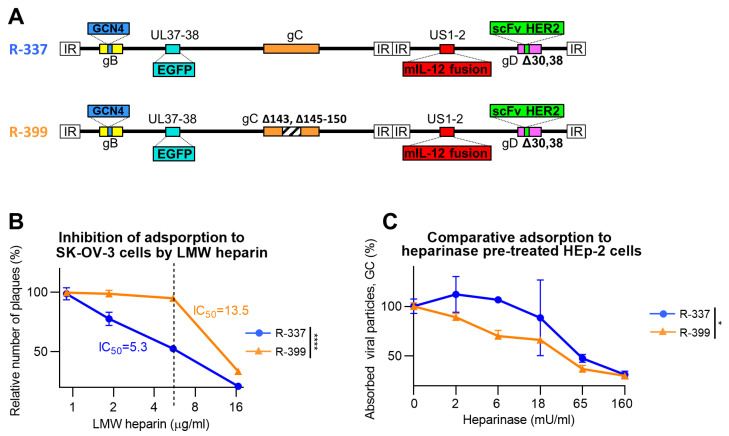
Adsorption of R-399 ReHV and of the parental R-337 to in vitro cultured cells. (**A**) Schematic representation of the genome of R-337 and its derivative R-399 that harbors a partial deletion of the heparan–sulfate-binding domain in the glycoprotein C (gC) gene. Indicated are the genetic loci of gC (wild type in R-337 and modified in R-399), gB with GCN4 insertion, gD with modifications for the detargeting from HSV-1 natural receptors Herpesvirus Entry Mediator (HVEM) and nectin1 and the retargeting to HER2, the insertion of murine interleukin 12 (mIL-12) gene in the Unique Short 1 (US1) and US2 intergenic locus, and of enhanced green fluorescent Protein (EGFP) in the Unique Long 37 (UL37) and UL38 intergenic locus. (**B**) Inhibition of virus adsorption to SK-OV-3 cells by low-molecular-weight (LMW) heparin, a heparan sulphate (HS) competitor. R-399 and R-337 viruses were pre-incubated with LMW heparin at the indicated concentrations for 1 h at 4 °C; the preincubated virions were then added to SK-OV-3 cells in the presence of the same amounts of competitor to allow virus adsorption. After the removal of non-adsorbed virus, cells were shifted to 37 °C to allow virus penetration and plaque formation. The number of plaques is expressed as percentage relative to the number of plaques in the heparin-untreated sample. The dashed line indicates the IC_50_ value for R-337. (**C**) Dose-dependent inhibition curve of R-399 or R-337 adsorption to HEp-2 pre-treated with Heparinase I to reduce the overall amount of cell surface HSs and CSs. Triplicate monolayers of HEp-2 cells were pre-treated with Heparinase I at the indicated final concentrations for 1 h at 37 °C. Cells were then put on ice; viral inocula were added to allow 1 h of adsorption at 4 °C. After inoculum removal and rinsing, the viral particles adsorbed to cells were quantified by qPCR by means of a probe annealing to the HSV DNA polymerase gene and expressed as percentage viral genome copies (GC), relative to the GC adsorbed to the heparinase-untreated control. (**B**,**C**) Each point (symbol) is the mean of triplicate samples ± SD. Statistical analysis was performed with the unpaired parametric *t*-test (two-tailed) on the values of area under the curve calculated for each biological replicate (normal distribution, equality of variances from Shapiro–Wilk test and F-test, respectively); results of the tests are reported in the graphs: * = *p*-value < 0.05; **** = *p*-value < 0.0001. Color codes: R-337 in blue, R-399 in orange.

The well-characterised HER2-retargted ReHV named R-337 [53] was selected as the starting virus to partially decrease the heparan sulphate and chondroitin sulphate-binding activity [53]. R-337 carries the EGFP reporter in the UL37-38 locus, the GCN4 peptide in gB for retargeting to a producer cell line, is detargeted from nectin1 and HVEM receptors through the deletion of amino acids 30 and 38 in gD and is armed with mIL-12 to boost its immunotherapeutic activity. We modified the nonessential glycoprotein gC that constitutes the major HS- and CS-binding glycoprotein in HSV [44] and left the HS-binding site in gB unaltered. Specifically, from gC, we deleted Arg143 and Arg145-Thr150, which were previously identified by Trybala et al. as containing critical amino acids for the HS- and CS-binding activities [48,49]. The resulting recombinant was named R-399; its genome is represented schematically in Figure 1A.

Firstly, we ascertained to what extent the genetic modifications in gC decreased R-399 adsorption to cells. Of note, while HSV entry into cells occurs at 37 °C or similar physiological temperatures, adsorption also occurs at 4 °C. Hence, the latter temperature is preferred in virus-attachment studies. The HS-mediated adsorption of HSV to cells can be competitively inhibited by soluble heparin, e.g., low-molecular-weight (LMW) heparin [56]. R-399 and, for comparison, the parental R-337 were preincubated with increasing amounts of LMW heparin and then allowed to adsorb to SK-OV-3 cells for 2 h at 4 °C in the presence of the inhibitor. The virus inoculum was then removed and the temperature was shifted to 37 °C to allow the adsorbed virus to infect the cells and form plaques. Figure 1B reports a typical dose-dependent inhibition experiment for the two viruses and shows that the 50% inhibitory concentration (IC_50_) was about three-fold higher for R-399 than for R-337. The comparative determination of the IC_50_ was calculated on the percentage of plaques relative to the number of plaques in the LMW heparin-untreated sample. It should be noted that the genome copies (GCs) GC/PFU ratio differed between R-399 and R-337. In the experiment shown in Figure 1B, the GC/PFU ratios were 3211 and 1798 for R-399 and R-337, respectively. For the virion stocks employed in subsequent experiments, the mean ratio was 3326 and 1561 for R-399 and R-337, respectively. These ratios indicate that R-399 was mildly defective in comparison to R-337 in terms of infectious dynamics in cell cultures, as expected.

To further highlight defects in R-399 attachment to cells, we pretreated cells with Heparinase I. The pretreatment decreases the amount of HS on the cell surface and facilitates the evaluation of impairments in virus attachment [49]. We employed the HER2-negative HEp-2 cells so as to avoid the subsequent interaction of the recombinant gD present in R-399 and R-337 with the cell surface. Specifically, when the combination of wt-HSV and nectin1-positive cells or of HER2-retargeted/nectin1-detargeted HSV and HER2-positive cells are analyzed, virus adsorption to cells is mediated by both the gC interaction with GAGs and the gD interaction with its receptor. Preliminarily, we determined the dose of heparinase that completely inhibited R-337 attachment to cells and found it to be ≤ 250 mU/mL (Figure S1). We inferred that such conditions substantially removed HS from the surface of HEp-2 cells, and in subsequent experiments, we employed lower heparinase concentrations, ranging from 0 to 160 mU/mL. R-337 or R-399 were then allowed to adsorb to the heparinase-pre-treated HEp-2 cells. Figure 1C shows that at low heparinase concentrations (left-hand part of the diagram), i.e., under conditions of partial removal of HS moieties from the cell surface, the adsorption of R-399 was less efficient than that of R-337. As expected, at the highest heparinase concentrations, i.e., under conditions that fully blocked R-337 adsorption to cells in the preliminary experiment, both viruses failed to adsorb to cells. The two series of experiments shown in Figure 1B,C concordantly document that the removal of key residues in the HS-binding site in gC decreased the R-399 ability to adsorb to cells via cell-surface HSs. Since the binding site to CSs in gC overlaps with the binding site to HSs [48], R-399 is also likely defective in the interaction with CSs.

### 3.2. Characterisation of Replicative Ability of R-399

**Figure 2:**
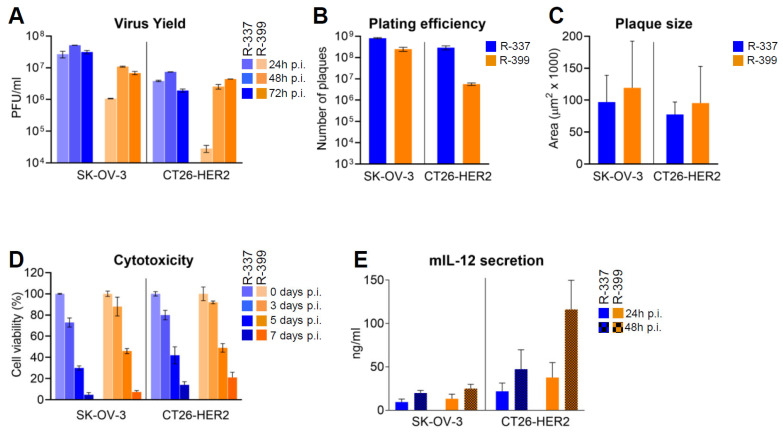
In vitro characterisation of replicative ability of R-399. (**A**) Time course of R-337 and R-399 replication in SK-OV-3 and CT26-HER2 cells. Triplicate monolayers of the indicated cells were infected at an input multiplicity of infection of 0.1 PFU/cell, as titrated in the respective cell line and harvested at 24, 48, and 72 h after infection. Progeny virus was titrated in triplicate in SK-OV-3 cells. Each column represents the mean titer ± S.D. (**B**) Efficiency of R-337 or R-399 infection (plating efficiency) in SK-OV-3 and CT26-HER2 cell lines. For each virus, a suspension containing approximately 5 × 10^8^ PFU/mL, and serial 1:10 dilutions thereof, were plated on triplicate monolayers of SK-OV-3 or CT26-HER2 cells; the number of plaques scored in the two different cell lines after 5 d is reported. Each column shows the mean number of plaques ± S.D. (**C**) Mean size of plaques formed by R-337 or R-399 in SK-OV-3 or CT26-HER2 cell monolayers. Samples were the ones described in Panel B. At the end of the experiment, 20 plaques for each virus were subjected to plaque-size determination by means of Nis Elements–AR–Imaging Software; plaque size was expressed as µm^2^. Each column represents the mean size of plaques in the indicated sample ± S.D. (**D**) Time course of cytotoxic effect exerted by R-337 or R-399 in SK-OV-3 and CT26-HER2 cells. For each time point, quadruplicate monolayers were infected at 0.1 PFU/cell. Cytotoxicity was determined by alamar blue viability and expressed as the percentage of non-viable cells relative to uninfected viable cells. Each column represents the mean of two independent experiments ± S.D. (**E**) Secretion of mIL-12 by SK-OV-3 and CT26-HER2 cells infected with R-337 or R-399 recombinants. mIL-12 was quantified by ELISA with the aid of the mouse ELISA kit, relative to a standard curve. Each column represents the mean of three independent experiments where each sample was run in duplicate ± S.D. Color codes: as in Figure 1.

In this series of experiments, R-399 was characterised in vitro to evaluate the biological consequences of reduced GAG binding. A typical experiment reporting the time course of R-399 replication in human HER2-positive SK-OV-3 cells and in the murine CT26 tumor cells transgenically expressing human HER2 (CT26-HER2) is shown in Figure 2A. The growth of R-399 was about half a log lower than that of R-337 in SK-OV-3 cells; in CT26-HER2 cells, it was delayed, which was consistent with the mild defects in infection dynamics seen in Figure 1 and reached substantially similar titers at 48–72 h after infection.

The relative efficiency of R-337 and R-399 to infect SK-OV-3 or CT26-HER2 cell lines (also referred to as plating efficiency) was quantified as the number of plaques formed by a fixed amount of each virus plated onto monolayers of SK-OV-3 or CT26-HER2 cells (Figure 2B). A suspension of R-337 or of R-399 containing approximately 5 × 10^8^ PFU/mL, and serial dilutions thereof, were plated in SK-OV-3 or CT26-HER2 cell monolayers. The number of plaques was scored 5 days later. As shown in Figure 2B, whereas R-337 plated at similar efficiency in the two cell lines, R-399 plated at a two-log lower efficiency in CT26-HER2 cells, probably reflecting different amounts of cell-surface GAGs. In the same experiment, we further quantified the ability of the two viruses to spread from cell to cell as inferred from the plaque sizes. Figure 2C shows that R-399 did not significantly differ from R-337 with respect to cell-to-cell spread in either cell line.

Figure 2D shows the kinetics of R-337 and R-399’s ability to kill infected SK-OV-3 and CT26-HER2 cells, measured as alamar blue viability. There were no differences between R-399 and R-337 in either SK-OV-3 or CT26-HER2 cells.

Lastly, we compared the extent of mIL-12 expression from SK-OV-3 or CT26-HER2 cells infected by R-399 or R-337. Figure 2E shows that there was no major difference between the two viruses. The amounts of mIL-12 were somewhat higher at 48 h than at 24 h, as expected. The higher secretion from CT26-HER2 cells probably reflected the higher number of cells in the culture.

Altogether, the results indicate that R-399’s ability to adsorb to cells (Figure 1B,C) was reduced relative to that of R-337’s. This reduction likely affected the initial dynamics of infection of CT26-HER2cells, and yet it did not grossly impair R-399’s ability to replicate, spread from cell to cell, cause cytotoxicity, and secrete mIL-12.

### 3.3. Blood Clearance and Biodistribution of Systemically Administered R-399

As mentioned, we expected that the R-399 defects in interactions with GAGs, coupled with the inability to interact with nectin1 and HVEM expressed in endothelial cells and blood cells, would result in a longer half-life of circulating R-399 and an increased likelihood to populate tissues upon leaving the bloodstream.

**Figure 3:**
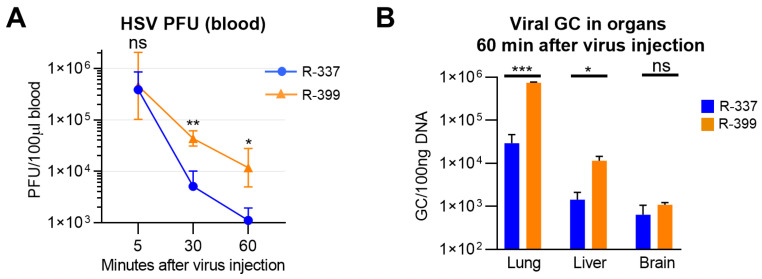
Clearance and biodistribution of systemically administered R-399 and parental R-337. (**A**) Kinetics of R-399 and R-337 blood clearance. huHER2-trangenic mice (herein BALB/c-TG) (*n* = 10) mice were administered via an IV injection with 1 × 10^6^ CT26-HER2 cells to establish metastatic-like lung tumors and, 14 d later, they received a single IV injection of R-337 (*n* = 5) or R-399 (*n* = 5) (1 × 10^7^ PFU/mouse). Blood samples were collected 5, 30, and 60 min after injection; infectious virus in the blood samples was titrated in SK-OV-3 cells and expressed as PFU/100 µL blood. (**B**) Biodistribution to indicated tissues of R-399 or R-337 were expressed as viral genome copies (GCs)/100 ng of tissues DNA. Lung, liver, and brain samples (*n* = 4 for each virus) were collected 60 min after virus injection and homogenized, and the total DNA was purified as detailed in Section 2. The content in viral GCs was quantified by qPCR using a probe for HSV DNA polymerase and a standard curve to interpolate the results. Data were expressed as GCs/100 ng of DNA. (**A**,**B**) Symbols and columns indicate the mean values from the indicated number of mice ± SD. Statistical analysis was performed (**A**) by means of the unpaired nonparametric two-tailed Mann–Whitney test (non-normal distribution from Shapiro–Wilk test) for each time point, and (**B**) by means of the unpaired parametric two-tailed *t*-test with Welch’s correction for each organ (normal distribution, non-equal variances from Shapiro–Wilk test and F-test, respectively). Results of the statistical analyses are reported in the graphs: * = *p*-value < 0.05; ** = *p*-value < 0.01; *** = *p*-value < 0.001; “ns” = no significant difference. Color codes: as in Figure 1.

R-399 was administered via an IV injection to mice bearing CT26-HER2 lung tumors generated upon IV administration of the cancer cells. Blood samples were collected at 5, 30, and 60 min. Lung, liver, and brain tissues were harvested at sacrifice (60 min time point). Figure 3A shows that blood clearance of infectious R-399 was indeed delayed relative to that of R-337 and resulted in one log increase in circulating infectious virus at 60 min. Virus burden in tissues was quantified as genome copies, which is a more sensitive evaluation than the titration of the infectious virus due to the high GC/PFU ratio. The R-399 burden was higher than that of R-337 in lungs and liver, but not in the brain (Figure 3B). These results suggest an increased uptake of R-399 from blood by tumor-positive lungs and by liver. The higher distribution of R-399 to the liver is consistent with the removal and breakdown of the virus and the blood-cleansing functions of this organ. The results document higher persistence in blood and improved delivery to organs of systemically administered R-399 relative to R-337. The biodistribution of R-337 was consistent with what was recently reported by our group [22].

### 3.4. Comparative Efficacy of Systemically Administered R-399 and R-337 against Metastatic-like Lung Tumors

**Figure 4:**
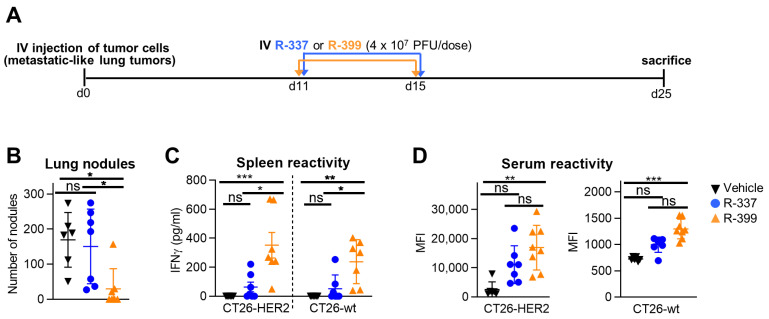
Efficacy of R-399 and R-337 monotherapy on the growth of multiple metastatic-like lung CT26-HER2 tumors. (**A**) Treatment scheme with IV virus injections to treat multiple metastatic-like lung tumors. 3 × 10^5^ CT26-HER2 cells were administered via an IV injection in BALB/c-TG mice to establish metastatic lung tumors. Eleven and 15 days later, mice received two IV injections of R-337 or R-399 (4 × 10^7^ PFU/dose). Mice were sacrificed 25 days after tumor cell implantation; lungs were harvested, perfused with the staining solution, and fixed in Fekete’s solution. Lung metastatic nodules were counted under a microscope. (**B**) The number of tumor nodules scored in each mouse at lung surface. (**C**,**D**) Immune responses to CT26-wt and CT26-HER2 tumor cells in splenocytes (**C**) were detected as IFNγ secretion upon co-culture, and in sera (**D**), they were detected as mean fluorescence intensity (MFI) reactivity to the indicated cells. (**B**–**D**) Each symbol corresponds to an individual mouse; the horizontal line indicates the mean value. Vertical bars represent ± SD. Statistical analysis was performed with the nonparametric Kruskal–Wallis test with Dunn’s correction (non-normal distribution from Shapiro–Wilk test). Within each analysis, all possible comparisons between the study groups were considered; in (**C**,**D**), separate analyses were conducted for CT26-HER2 and CT26-wt samples. Results of the tests are reported in the graphs: * = *p*-value < 0.05; ** = *p*-value < 0.01; *** = *p*-value < 0.001; “ns” = no significant difference. Mice treated with R-337 (*n* = 7) in blue, R-399 (*n* = 7) in orange, or vehicle (*n* = 6) in black.

To compare the anticancer efficacy of R-399 and R-337, mice were engrafted via an IV injection with CT26-HER2 cells; by this route, the cancer cells allow for the rise of tumor nodules in the lungs, which are considered a model of metastatic disease [22]. R-399 or R-337 was administered via an IV injection on Days 11 and 15, 4 × 10^7^ PFU/dose. Mice were sacrificed on Day 25 (schedule in Figure 4A). The amount of R-337 was chosen based on previous experiments to achieve a partial anticancer effect and better emphasize any difference between the two viruses [22]. The efficacy results were expressed as the number of superficial lung nodules. Figure 4B shows that R-337 reduced the number of tumor nodules in three out of seven mice. R-399 produced an almost complete response in six out of seven mice. The difference between the anticancer effect of R-399 and R-337 was statically significant (Figure 4B). The treatment elicited a T-cell response that was higher in mice treated with R-399; this response was addressed to both the HER2 antigen and the agnostic antigens of CT26-wt cells (Figure 4C). Mice also mounted a strong antibody response to CT26-HER2 cells and a weak response to CT26-wt cells; again, the B-response was higher in the R-399 arm (Figure 4D). Altogether, upon systemic administration, R-399 was superior to R-337 in anticancer efficacy and in eliciting T and B responses.

### 3.5. Comparative Efficacy of Intratumorally Administered R-399 and R-337 against Subcutaneous Tumors

**Figure 5:**
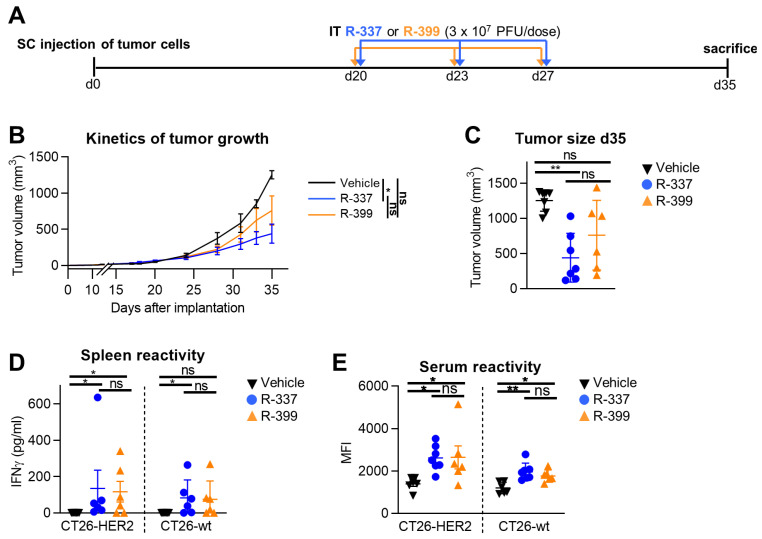
Efficacy of R-399 and R-337 monotherapy on the growth of subcutaneous CT26-HER2 tumors. (**A**) Treatment scheme with IT virus injections to treat the subcutaneous tumors. BALB/c-TG mice were implanted SC with 1 × 10^6^ CT26-HER2 cells. Twenty days later, when the tumor volume averaged 70–100 mm^3^, mice received 3 IT injections of R-337 (*n* = 7), R-399 (*n* = 6), (3 × 10^7^ PFU/dose), or vehicle (*n* = 7), administered at 3–4-day intervals. (**B**) Cumulative tumor growth curves for CT26-HER2 tumors. Each point represents the mean value relative to six-to-seven mice. (**C**) Tumor volumes at Day 35 after implantation. (**D**,**E**) Immune responses to CT26-wt and CT26-HER2 tumor cells in splenocytes (**D**) detected as IFNγ secretion upon co-culture, and in sera (**E**) detected as mean fluorescence intensity (MFI) reactivity to the indicated cells. (**C**–**E**) Each symbol corresponds to an individual mouse; the horizontal lines indicate the mean values; vertical bars indicate ± S.D. (**B**–**E**) Statistical analysis was performed with (**B**,**C**,**E**) the ordinary one-way ANOVA with Tukey’s correction (normal distribution, equality of variances from Shapiro–Wilk test and Bartlett’s test, respectively) or (**D**) the nonparametric Kruskal–Wallis test with Dunn’s correction (nonnormal distribution from Shapiro–Wilk test). (**B**–**E**) Within each analysis, all possible comparisons between the study groups were considered; in (**D**,**E**), separate analyses were conducted for CT26-HER2 and CT26-wt samples. (**B**) For each tumor-growth curve, the values of the area under the curve were calculated and compared in the statistical analysis. Results of the tests are reported in the graphs: * = *p*-value < 0.05; ** = *p*-value < 0.01; “ns” = no significant differences. Color code as in Figure 4.

The higher efficacy of IV-administered R-399 over R-337 might be due to a higher amount of R-399 reaching the tumors as a consequence of the differences in biodistribution seen in Figure 3, or to a superior intrinsic performance of R-399 within the tumor bed. To discriminate between these two possibilities, we compared the anticancer efficacy of the two viruses upon IT administration. Mice were SC engrafted with CT26-HER2 tumors and treated via an IT injection with three doses each of R-399 or R-337 at Days 20, 23, and 27 (Figure 5A). Mice were sacrificed when tumors in the control arm reached the end-point volumes. Figure 5B,C shows that R-337 inhibited tumor growth, which is in agreement with previous results [53]. R-399 performed worse than the parental R-337 and exerted a moderate antitumor effect. Both viruses elicited similar T and B cell responses to CT26-HER2 cells and CT26-wt cells (Figure 5D,E). Hence, in the IT setting, R-399 was not superior but somewhat inferior to R-337. We conclude that the superior efficacy observed in the systemic administration setting was a consequence of the higher amount of R-399 that reached the lung tumors.

## 4. Discussion

It is well established that the interaction of HSV gC, and less so of gB, with GAGs, in particular, with HSs and, to a lesser extent, CSs, provides the initial virus attachment to cultured cells [44,48,49]. The contribution of the gC–GAGs interaction during in vivo infection is not known. We reasoned that the gC interaction with GAGs, which are broadly expressed in endothelial cells, blood cells, and tissues, contributes to the subtraction from the circulation of systemically administered oHSVs, including ReHVs. Our aim was to slow down blood clearance, increase delivery to the tumors, and, consequently, enhance the anticancer efficacy of systemically administered HER2-retargeted ReHV by combining the decrease in gC-mediated binding to GAGs with the specific retargeting to cancer receptors intrinsic to ReHV technology. The latter ensures that any intravenously administered circulating ReHV does not interact with the natural HSV entry receptors, nectin1 or HVEM, and, thus, further reduces virus adsorption to off-target cells, including endothelial cells and blood cells.

As a preliminary approach to impair the gC–GAG interaction, we genetically modified gC in R-337, a HER2-retargted ReHV, and deleted a sequence harboring seven amino acids (Arg143, Arg145-Thr150) critical for this interaction [48,49]. The substitution of two of them was reported to reduce the attachment of wt-HSV to heparinase-treated HEp-2 cells by about 80–90% [49]; the heparinase treatment decreased the amount of cell surface HSs and, thus, favored the detection of the gC-defective phenotype. The gC modification in the resulting recombinant named R-399 indeed reduced its attachment to cell and also increased the IC_50_ of the competitive inhibitor heparin. Of note, while our results formally prove that R-399 was defective in the interaction with HSs, the notion that the very gC region mutated in R-399 is also critical for binding to CSs [48] makes it likely that R-399 was also defective in the interaction with CSs.

In vivo, the gC modification increased the blood half-life of systemically administered R-399, as well as its biodistribution to tumor-positive lungs and to tumor-negative liver. Ultimately, it greatly increased the efficacy of systemically administered R-399 against lung tumors, a model of metastatic disease. Remarkably, it did not increase the efficacy of intratumorally administered R-399, which was somewhat lower than that of intratumorally administered R-337, which is in agreement with the mild defects in infection dynamics seen in cultured cells. These results provide evidence that the increased efficacy of R-399 relative to R-337 upon systemic administration was dependent on increased half-life in circulation and biodistribution to tissues.

To our knowledge, this is the first in vivo evidence that a partial impairment in the interaction of gC with HSs, and possibly with CSs, results in the prolonged half-life of circulating ReHV, an increased amount of systemically administered ReHV that becomes adsorbed to tissues, and, ultimately, an improved anticancer efficacy of systemically administered ReHV. We note that the engineered gC modification resulted in a rather moderate reduction in the in vitro attachment of R-399 to SK-OV-3 and CT26-HER2 cells. There is sufficient know-how in the field to further reduce such interactions through additional mutations in gC [49] and, if needed, in the HS-binding site in gB. Altogether, the current paper and the other paper from our lab in this special issue [22] provide tools on how to counteract obstacles to the systemic delivery of ReHVs.

## 5. Conclusions

The systemic delivery of oncolytic viruses is desirable for hard-to-reach or metastatic cancers, which altogether represent high-impact unmet clinical needs. This route of administration is hampered by several factors, especially for the OVs derived from viruses seroprevalent in the human population. In an accompanying paper, we analyzed blood factors like complementary and virus-neutralizing antibodies [22]. Here, we analyzed to which extent virus adsorption to off-tumor and off-target tissues acts as a sink and subtracts ReHV from circulation. We combined the decrease in gC-mediated virus attachment to off-tumor tissues via GAGs with the specific retargeting to cancer receptors intrinsic to ReHV technology.

We report that the gC modification increased the blood half-life of systemically administered R-399, its biodistribution to tumor-positive lungs and tumor-negative livers, and, ultimately, the efficacy of systemically administered R-399 against lung tumors. In contrast, the efficacy of intratumorally administered R-399 was essentially very similar to that of IT-administered R-337, arguing that the higher efficacy upon IT administration was the effect of a higher amount of virus reaching the tumor. To our knowledge, this is the first in vivo evidence that a partial impairment of the gC interaction with heparan sulphates, and possibly with chondroitin sulphates, impacts the half-life of circulating virus and results in improved anticancer efficacy of systemically administered ReHV.

In the infection of wt-HSV, a gC-mediated attachment is immediately followed by the interaction of gD with nectin1 or HVEM receptors, which are broadly expressed in off-tumor tissues and provide high-affinity binding of HS virions to cells. Current findings raise the question of whether an impairment in the gC–GAG interaction can improve the efficacy of systemically administered oHSVs other than ReHVs and, ultimately, of any OV that interacts with GAGs. In the case of oHSVs that have maintained wild-type virus tropism, in vitro studies with soluble molecules suggest that virus adsorption to GAGs and the interaction of gD with its natural receptors nectin1 or HVEM occur at comparable affinities [58,59,60]. Likely, the gD interaction with its natural receptors provides an additional anchorage of HS virions to nectin1- or HVEM-positive cells. Whether a decrease in GAG binding would improve the systemic delivery of oHSVs other than ReHVs was beyond the purpose of the current study and remains to be investigated.

## Data Availability

All the data presented in this study are available in the article.

## Supplementary Materials

The following supporting information can be downloaded at: https://www.mdpi.com/article/10.3390/cancers16061143/s1, Figure S1: Inhibition of virus absorption to HEp-2 cells with reduced amounts of HS and CS.
