# Supplementary material for: Decrease in Heparan Sulphate Binding in Tropism-Retargeted Oncolytic Herpes Simplex Virus (ReHV) Delays Blood Clearance and Improves Systemic Anticancer Efficacy
Source: Cancers (Basel). 2024 Mar 13;16(6):1143. doi: 10.3390/cancers16061143 (PMC10969551; doi:10.3390/cancers16061143)
Supplement: Supplementary file 1 [file cancers-16-01143-s001.zip › cancers-2844135-supplementary.pdf]

cancers MDPI

# Supplementary Materials: Decrease in Heparan Sulphate Binding in Tropism-Retargeted Oncolytic Herpes Simplex Virus (ReHV) Delays Blood Clearance and Improves Systemic Anticancer Efficacy

**Andrea Vannini, Federico Parenti, Cristina Forghieri, Gaia Vannini, Catia Barboni, Anna Zaghini, Tatiana Gianni and Gabriella Campadelli-Fiume**

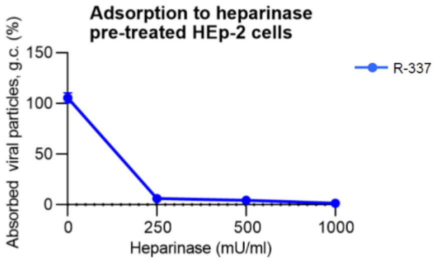


**Figure S1.** Inhibition of virus absorption to HEp-2 cells with reduced amounts of HS and CS. Details as Fig. 1C legend.
